# Social Isolation and Loneliness: Strengthening Education and Training

**DOI:** 10.1093/geroni/igaa057.2514

**Published:** 2020-12-16

**Authors:** Tracy Lustig

**Affiliations:** The National Academies of Sciences, Engineering, and Medicine, Washington, District of Columbia, United States

## Abstract

This paper reviews recommendations for a variety of opportunities to improve education and training of the health care workforce on the health impacts of SIL and clinical approaches for assessment as well as testing different approaches for such education and training. The fifth goal of the NASEM study is to “strengthen ties between the health care system and community-based networks and resources.” Similar to other social determinants of health, addressing SIL will require coordinated efforts among a variety of stakeholders. This paper reviews recommendations for improving coordination, including team-based care and promotion of tailored community-based services, as well as the creation of a centralized repository for new evidence and best practices. Finally, connections between this NASEM study and the 2019 report Integrating Social Care into the Delivery of Health Care are discussed. Part of a symposium sponsored by Loneliness and Social Isolation Interest Group.

